# ABSTRACT THOUGHT LEADS TO UNPARALLELED BEAUTY: A STUDY ON BULLSHIT RECEPTIVITY AND SCAM SUSCEPTIBILITY

**DOI:** 10.1093/geroni/igae098.0366

**Published:** 2024-12-31

**Authors:** Stacey Wood, Sagrika Jawadi, David Hengerer

**Affiliations:** Scripps College, Claremont, California, United States; Scripps College, Claremont, California, United States; Claremont Graduate University, Claremont, California, United States

## Abstract

In the age of disinformation, the ability to discern the credibility of information is more vital than ever. To assess an individual’s susceptibility to pseudo-profound statements, or “bullshit”, the Bullshit Receptivity (BSR) Scale, a list of meaningless, mundane, and profound statements, was devised in 2015. In Study 1, a lifespan sample of 213 individuals (age range 24-78, M = 46.05, SD = 17.21) participated in the study via MTurk and were asked to rate the profundity of various bullshit and mundane statements, complete a vocabulary test, and complete a subset of the CART (rational thought). Older adults performed significantly better than did younger and middle-aged adults on the overall BSR scale. The relationship between age and BSR score was partially mediated by performance on the vocabulary test, with a greater vocabulary leading to lower susceptibility to bullshit (β = -.0248, p <.001, SE =.002). In Study 2, we introduced scam susceptibility to investigate the relationship between scam susceptibility and bullshit receptivity. We replicated the study in person with 92 neurotypical participants (M = 19.96, SD = 5.42). Participants also completed subtests from the NIH Toolbox Cognition Battery and rated the appeal of fraudulent investment pitches and red-flag persuasion tactics. Scam susceptibility was positively related to bullshit receptivity, as measured by rated profundity of pseudo-profound statements (r =.27, p <.05). This information can be used to better understand the cognitive underpinnings of scam susceptibility, especially among older adults who are targeted for scams.

